# Origins of submovements in movements of elderly adults

**DOI:** 10.1186/1743-0003-5-28

**Published:** 2008-11-13

**Authors:** Laetitia Fradet, Gyusung Lee, Natalia Dounskaia

**Affiliations:** 1 Movement Control and Biomechanics Laboratory, Arizona State University, Tempe, AZ 85287, USA

## Abstract

**Background:**

Slowness is a well-recognized feature of movements in aging. One of the possible reasons for slowness suggested by previous research is production of corrective submovements that compensate for shortened primary submovement to the target. Here, we re-examine this traditional interpretation and argue that the majority of submovements in older adults may be a consequence rather than the cause of slowness.

**Methods:**

Pointing movements in young and older adults were recorded. Conditions for submovement emergence were manipulated by using small and large targets and three movement modes: discrete (required stopping on the target), reciprocal (required reversal on the target), and passing (required crossing the target and stopping after that). Movements were parsed into a primary and secondary submovement based on zero-crossings of velocity (type 1 submovements), acceleration (type 2 submovements), and jerk (type 3 submovements). In the passing mode, secondary submovements were analyzed only after crossing the target to exclude that they were accuracy adjustments.

**Results:**

Consistent with previous research, the primary submovement was shortened and total secondary submovement incidence was increased in older adults. However, comparisons across conditions suggested that many submovements were non-corrective in both groups. Type 1 submovements were non-corrective because they were more frequent for large than small targets. They predominantly emerged due to arm stabilization and energy dissipation during motion termination in the discrete and passing mode. Although type 2 and 3 submovements were more frequent for small than large targets, this trend was also observed in the passing mode, suggesting that many of these submovements were non-corrective. Rather, they could have been velocity fluctuations associated predominantly with low speed of movements to small targets.

**Conclusion:**

The results question the traditional interpretation of frequent submovements in older adults as corrective adjustments. Rather, the increased incidence of submovements in older adults is directly related to low movement speed observed in aging, whereas the relationship between submovement incidence and target size is a result of speed-accuracy trade-off. Aging-related declines in muscular control that may contribute to the disproportional increases in submovement incidence during slow movements of older adults are discussed.

## Background

Slowness is one of the most robust effects of aging on movement performance. Decreases in movement speed for 30%–70% of older adults compared with young adults have been demonstrated on a variety of motor tasks [1-10]. Pointing and reaching tasks have been exploited most frequently to investigate reasons for movement slowing with aging. In addition to decreased peak velocity and prolonged deceleration phase, a shortened primary submovement and performance of secondary submovements have been considered contributing factors to movement slowness in elderly.

The primary submovement represented by the smooth, bell-shaped velocity profile has been interpreted as a ballistic movement portion driven by the initial control plan. It is assumed that inaccuracy of the initial control plan and neuromuscular noise during motion may cause deviations of the primary submovement from the target. Accordingly, secondary submovements, i.e. small irregularities that often emerge in the final movement portion, have been viewed as corrective adjustments performed to improve movement accuracy [11-18]. Since neuromuscular noise increases with aging, the shortened primary submovement in older adults has been accounted for as a compensatory strategy employed by these subjects to decrease variability of the initial, ballistic portion of movement, and to increase pointing accuracy by performing small corrective submovements [2,19-24]. This interpretation is supported by an observation that decreases in target size are accompanied by shortening of the primary submovement and by more frequent emergence of secondary submovements.

Recent studies have challenged the traditional interpretation of the role of submovements in movements of young adults [25-27]. These studies suggest that secondary submovements may be not corrective adjustments but rather represent irregular fluctuations in the velocity profile emerging from different reasons. By using the same method [16] as in many studies that developed the traditional interpretation, submovements were distinguished in [25-27] with analysis of zero-crossings in the velocity (type 1 submovements), acceleration (type 2 submovements), and jerk (type 3 submovements) profiles. It was found that the majority of type 1 submovements, and in some conditions type 2 submovements, were non-corrective. They represented fluctuations emerging during motion termination and stabilization of the limb at the target. These submovements emerged more frequently during movements to large than small targets, i.e. when movement speed was higher. Other submovements, predominantly of type 3, appeared more frequently during movements to smaller targets. Nevertheless, evidence suggested that some of these submovements may also have been non-corrective velocity fluctuations emerging due to low movement speed that is usually observed for small targets [28].

The purpose of the present study is to investigate whether the finding obtained for young adults that many submovements are not corrective but are a by-product of motion termination and low movement speed [25-27] is applicable to submovements in older adults. In this case, the contribution of corrective submovements to slowness in aging suggested by the traditional interpretation of submovements would need to be re-considered. Indeed, the increased frequency of submovements in older adults should then be interpreted as a consequence rather than a cause of movement slowness in aging.

A difficulty related to investigation of submovement origins is that submovements emerging from distinct sources have the same kinematic properties, and therefore, they cannot be distinguished with a kinematic analysis. Indeed, methods of submovement detection that have been used, such as finding zero-crossings of the velocity, acceleration, and jerk [16] or fitting the velocity profile with a series of bell-shaped functions [29-31] detect submovements regardless of their origin. To overcome this difficulty and examine sources of submovements in older adults, we exploit the approach of [25,26,28] that uses manipulations of movement conditions to emphasize the production of submovements of distinct origins. In these studies, the contribution of motion termination to submovement production was established by comparing incidence of the three submovement types between discrete movements that stopped and dwelled on the target and reciprocal movements that reversed at the target without dwelling. As justified in detail in [25], discrete movements include a special component of control, motion termination, that dissipates kinematic energy and arrests the arm, stabilizing it at the target. In contrast, reciprocal movements performed without dwelling on the target do not include motion termination because the stabilization of the arm at the target is not performed.

In addition to the movement mode manipulations, target size was manipulated in those studies to emphasize the role of accuracy requirements on submovement production. It was found that type 1, and sometimes type 2 submovements were frequent during the discrete mode and they were almost absent during the reciprocal mode. Also, incidence of these submovements increased with increases in target size. Based on these findings, it was concluded that these submovements were not corrective but were caused by motion termination and stabilization of the limb at the target.

Type 3 submovements were observed equally in the discrete and reciprocal movements and were more frequent during movements to small than to large targets. These characteristics of type 3 submovements are in agreement with the traditional interpretation of them as corrective adjustments. However, it was found that during cyclical movements of different frequency levels, incidence of type 3 submovements depended on frequency level and did not depend on target size [26]. This finding suggests that type 3 submovements (at least, the majority of them) may also be not corrective. Instead, they may be irregular velocity fluctuations emerging primarily during slow movements.

A support for this interpretation was provided by including in the experiment a passing mode in addition to the discrete and reciprocal modes [27]. In the passing mode, subjects were instructed to cross the target and terminate motion after that. Movements performed in the passing mode were like wiping with a sweeping motion of the finger. Apparently, submovements that emerged after crossing the target were not corrective adjustments, since the target had already been passed, and no restrictions were imposed on the location for movement termination that could elicit corrective adjustments. It was found that type 3 submovements consistently emerged after the target had been crossed, and their incidence increased with decreases in target size. This result demonstrates that the inverse relationship between type 3 submovement frequency and target size is not necessarily a feature of corrective submovements. An alternative interpretation discussed in [27] is that type 3 submovements emerge more frequently when movement speed is lower, as it takes place in movements to smaller targets.

To investigate whether movements of older adults include non-corrective submovements of the same origins as those found in young adults, the experimental paradigm developed in [27] is used here. Namely, submovements are studied in young and older adults during pointing movements performed in three modes, discrete, reciprocal, and passing. In addition, target size was manipulated to emphasize the influence of accuracy requirements on submovement production.

## Methods

Methods were similar to those described in [27].

### Participants

Sixteen older adults (12 males, 4 females, mean age 72.4 years, SD = 6.4 years) and a control group of sixteen young adults (10 males, 6 females, mean age 24.7 years, SD = 4.9 years) participated in the experiment. All subjects were right-handed. After an explanation of the experiment, subjects signed informed consent approved by the Human Subjects Institutional Review Board (IRB) of Arizona State University. All participants met study criteria as follows: normal or corrected vision, and the presence of full range of motion in the finger, wrist, and elbow joints, and functional range of motion in the shoulder joint. In addition, older adults met a cut-off score of 25 on the Mini-Mental State Exam [32]. Also, older adults did not have a history of any central nervous system (CNS) disease.

### Procedure

Subjects sat comfortably in front of a Wacom Intuos (12 × 18) digitizer positioned on the top of a horizontal table. The height of the table was adjusted to provide right arm movements in the horizontal plane above the table. Movements were performed predominantly with rotations of the shoulder and elbow joints. The trunk position was restricted by the chair-back and the front edge of the table. The wrist was immobilized with a brace. The index finger was stretched and a pen was attached beneath it with low-friction Velcro tape. To prevent fatigue due to the effect of gravity, the upper arm was supported by a sling. Subjects moved the pen on the surface of the digitizing tablet from a home position to one of four targets. The home position was located 34 cm from the trunk on the body midline. The targets were placed at 20 cm distance in different directions from the home position. Motion of the pen was represented by motion of a cursor on a vertical computer screen (24 inches) positioned at 70 cm in front of the subject. The home position and the targets were also shown on the screen.

The purpose of the usage of the four targets in different directions was to test whether the submovement production in older adults depends on the joint coordination pattern and is influenced by inter-segmental dynamics during motion. Each target required joint movements in a distinct coordination pattern. *Target 1 *required shoulder flexion only, *Target 2 *required elbow extension and shoulder flexion, *Target 3 *required elbow extension only, and *Target 4 *required elbow and shoulder extension. Thus, the target locations were adjusted to the lengths of the arm segments to provide the required patterns of joint movements. The sequence of target location for the pointing tasks was randomized across subjects. Subsequent analysis confirmed that the choice of target locations successfully provided the required joint coordination patterns. For instance, during the discrete mode, mean shoulder and elbow amplitude was 23° ± 5.7° and 1° ± 3.8°, respectively, for target 1, 28° ± 8.8° and 36° ± 7.2° for target 2, 2° ± 3.3° and 27° ± 4.3° for target 3, and 12° ± 2.8° and 13° ± 4.0° for target 4. These values were very similar during the reciprocal mode. Similar manipulations tested in young subjects did not reveal any influence of joint coordination on submovement production [25,26]. Likewise, no effect of target location was found in the present study for any of the two subject groups. The data from the four targets were therefore combined in all subsequent analyses.

The targets had a square shape and were of two sizes, *small *(1.0 × 1.0 cm) and *large *(3.5 × 3.5 cm). Three modes of pointing movements from the home position to the target were performed, *discrete*, *reciprocal*, and *passing*. Discrete movements ended in the target area. Reciprocal movements required reversal within the target without dwelling. Passing movements consisted of crossing the target and stopping within the digitizer boundaries. To prevent a sequential movement in the passing mode, first to the given target as a via-point and then to an imaginary target at which motion could be terminated, subjects were instructed to perform passing movements in a single action as if they were "wiping" the target with a sweeping action. Later analysis confirmed that the velocity profile had the bell shape observed during movements to a single target, and not a double-peak velocity profile typical of movements that proceed to the final target through a via-point [33,34]. This suggested that subjects did not have an "imaginary" target at the end of passing movements that could elicit corrective submovements. The digitizer boundaries were at least 18 cm from the target in each direction. The three movement modes and the two target sizes were randomized across subjects.

The three modes allowed us to distinguish submovements related to motion termination because motion termination was included only in discrete and passing and not reciprocal movement. Also, submovements related to motion termination were disassociated from possible corrective submovements in the passing mode during which motion termination and accuracy regulation were performed separately from each other: motion termination was performed at the end of movement and accuracy regulation was performed before crossing the target. In addition to submovements emerging due to motion termination, the passing mode provided a possibility to examine whether there are non-corrective submovements associated with decreases in target size. Indeed, submovements emerging after passing the target could not be corrective because the target had already been passed at the moment of the emergence of these submovements. The traditional interpretation of submovements as corrective adjustments is predominantly based on the observation that submovement incidence is in the inverse relationship with target size. If it is found that non-corrective submovements observed in the passing mode are also more frequent when the target is smaller, this result would demonstrate that the inverse relationship between target size and submovement incidence cannot be used to conclude that submovements are corrective.

Movements were initiated in response to a verbal signal. Although the instruction was to move to the target as fast as possible, there was an ultimate requirement to reach the target. This requirement was different from the instruction used in [25,26]. In those studies, accurate target achievement was encouraged but missing the target and terminating motion nearby was allowed. Since that type of accuracy requirements may not sufficiently enforce corrective submovements, here we used the ultimate requirement to reach the target. Namely, subjects had to terminate motion strictly within the target in the discrete mode, to reverse motion inside the target without dwelling in the continuous mode, and to cross the target area in the passing mode. If any of these requirements was not fulfilled, an auditory signal was produced to inform the subject that he/she failed to perform the task, and that the trial had to be repeated. These strict accuracy requirements encouraged production of corrective submovements. Only successful trials were retained for subsequent analysis to insure that the incidence of corrective submovements would not be reduced due to the failure to follow the accuracy requirements. This procedure provided optimal conditions for emergence of corrective submovements, suggesting that if corrective submovements are not frequent in these conditions, they would be even less plausible in other conditions. Prior to data recording, practice trials were performed in each condition until the subject demonstrated stable ability to perform the task, and unsuccessful trials were rare. Eight successful trials were recorded for each condition. Visual observations during the experiment suggested that not more than 1–2 trials were dropped from the analysis in each subject across all conditions due to missing the target, and this number was not different between young and older adults.

A computer program provided the control for valid task performance by verifying the following conditions. During the discrete mode, the pen tip velocity and acceleration had to be nullified within the target area and stay below 5% of the velocity peak for at least 150 ms. During the reciprocal mode, the pen had to reach the target with zero velocity. However, velocity could not stay below 5% of its peak for a period longer than 60 ms. During the passing mode, the pen had to cross the target area with velocity higher than 5% of maximal velocity achieved during the preceding movement portion.

### Data recording and analysis

Pen motion was recorded by the digitizer at a sampling frequency of 100 Hz. These data were employed to present motion on the computer screen. Motion analysis was performed using data collected with a three-dimensional, optoelectronic tracking system (Optotrak, Northern Digital) at 100 Hz. Four reflective markers were attached to the sternum, shoulder, elbow, and tip of the index finger. Data from the markers were used to control for joint movement patterns corresponding to the four target locations. Arm endpoint motion was analyzed with use of data from the fingertip marker. Velocity, acceleration, and jerk were computed as derivatives of fingertip displacement using a differentiation method that simultaneously smoothes data. In this method, the data are approximated within a sliding window with a quadratic polynomial. The coefficients of the quadratic polynomial were then used for calculating the derivative at the window's center [35]. Positive values of velocity corresponded to motion towards the target.

Movement initiation was determined with the following technique. First, the moment of time was found at which the unsigned velocity of the fingertip marker exceeded 5% of peak velocity after being below this threshold for at least 150 ms. Then, a backward-tracing algorithm was used to determine the last preceding moment at which signed velocity was zero. Similarly, the end of the discrete and passing movements was determined based on the moment of time at which unsigned velocity was lower than 5% of peak velocity and stayed under this threshold for at least 150 ms. The moment at which signed velocity became zero after crossing the 5% threshold was considered as the movement end. Only the movement from the home position to the target were analyzed during the reciprocal mode. To define the end of this movement portion, two peak velocities were detected, during the motion to the target and during the reversal stroke. Starting from the second peak velocity, a backwards-tracing algorithm was used to detect the last moment when the unsigned velocity dropped below 5% of the first peak.

**Figure 1:**
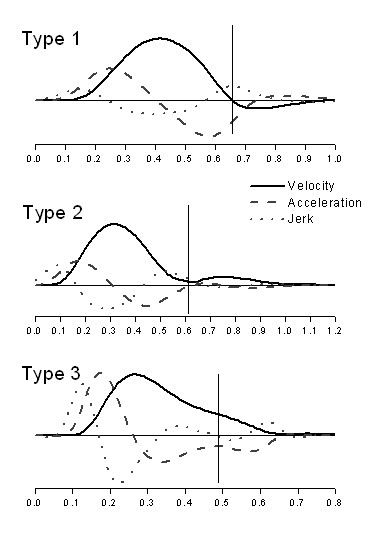
**Examples of submovements of type 1, 2, and 3**. Each panel shows the velocity, acceleration, and jerk profile during a discrete movement to a large target. The data were obtained from an older adult. The y-axes were different for the three profiles, and therefore, they are not shown for clarity of presentation. The vertical line marks a velocity zero-crossing from positive to negative values in case of the type 1 submovement, an acceleration zero-crossing from negative to positive values indicating the type 2 submovement, and a jerk zero-crossing from positive to negative values when the submovement was of type 3.

The end of the primary submovement within each movement to the target was distinguished with a method described in [16]. Although other methods of submovement detection have also been suggested [29-31], the majority of studies promoting the interpretation of secondary submovements as corrective adjustments employed the method of [16]. Since the goal of the present study was to re-examine this interpretation, we also used this method. The end of the primary submovement was identified by the first of any of the following events: a zero-crossing from positive to negative value occurred in the velocity profile (type 1 submovement); a zero-crossing from negative to positive value occurred in the acceleration profile (type 2 submovement); a zero-crossing from positive to negative value appeared in the jerk profile (type 3 submovement). Defined in this way, type 1 submovements corresponded to reversals in the trajectory, type 2 submovements represented re-accelerations towards the target, and type 3 submovements signified decreases in the rate of deceleration. Examples of the three submovement types during discrete movements are shown in Fig. 1.

Only secondary submovements emerging during the deceleration phase (i.e. that emerged after peak velocity) were analyzed, since corrective adjustments are likely to emerge during this phase. In addition, during the passing mode, only submovements that emerged after the target passing were analyzed. The target passing predominantly occurred after peak velocity, as reported in the Results section. Thus, not all submovements in the deceleration phase were analyzed in the passing mode but only those emerging after the target passing. By this way, we isolated submovements not related to accuracy regulation. The event of the target passing was determined as the time moment at which the distance between the fingertip and the target center started to increase.

If the end of the primary submovement did not coincide with the end of the entire movement, this movement was categorized as including a secondary submovement. Thus, the analysis focused only on the first interruption of the smooth velocity profile. Additional irregularities that may emerge in the later portion of the velocity profile were not included in the analysis as separate submovements because these irregularities may not be independent but influenced by the factor that causes the first velocity fluctuation. Accordingly, the movement portion between the end of the primary submovement and the end of the entire movement was for simplicity referred to as a secondary submovement.

Similar to previous studies that promoted the traditional interpretation of submovements, our analysis predominantly focused on submovement incidence, i.e. the portion of movements including secondary submovements among all movements in each condition. The previous studies usually did not separate the three types of submovements, but analyzed them together. However, many of the studies did not use all three types of submovements for analysis, focusing either on type 1 and 2, or on type 2 only, or on type 2 and 3. This divergence in the types of analysed submovements makes it difficult to compare results across the studies. For this reason, we analysed the three submovement types both together as it has been done in studies of other authors, and separately [25-27]. The separate analysis of the three submovement types is also justified by a consideration that different factors may cause different degrees of disturbance in the velocity profile represented by the three submovement types. This expectation has been supported by a finding that gross (type 1 and sometimes type 2) and fine (type 3) submovements had distinct sources [25-27]. Thus, in addition to the total incidence of submovements of all three types, incidence of each submovement type was also calculated for each condition and each subject as the number of movements with a secondary submovement divided by eight (the total number of movements performed in this condition). Accordingly, the sum of the incidences of the three submovement types was equal to the total submovement incidence.

### Statistical analysis

A 2 × 2 × 3 (group × target size × movement mode) repeated measures factorial analysis of variance (ANOVA) was applied to the majority of the computed characteristics. Group corresponded to older and young adults, target size corresponded to small and large targets, and movement mode corresponded to the discrete, reciprocal, and passing mode. Bonferoni post-hoc tests were conducted to perform pair-wise mode comparisons. The significance level was set at p < 0.05 for all analyses.

### Verification of the dependence of submovements on the filtering procedure

It was analyzed whether the specific method used in this study for differentiation and smoothing of the pen motion data influenced the emergence of the three types of submovements. With this purpose, results obtained for the total submovement incidence and submovement incidence by type with using this method were compared with the same characteristics obtained using two other smoothing methods and a MATLAB 2-point signal differentiation procedure. The first smoothing method was a 5^th^-order dual-pass low-pass Butterworth filter with a cut-off frequency of 7 Hz. The second method was a MATLAB cubic smoothing spline procedure *csaps*. Although using the different smoothing procedures resulted in slight variations in the values of submovement incidence in each condition, the statistically significant main effects and interactions were the same for all three methods. This demonstrated that the majority of submovements of all three types were not an artifact of the differentiation and smoothing procedure. Instead, they were inherent features of movement kinematics and their emergence depended on movement conditions, as described next.

## Results

### Peak velocity

**Figure 2:**
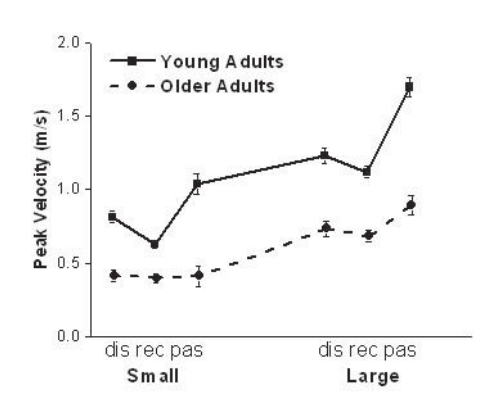
**Peak velocity**. Peak velocity during the discrete (dis), reciprocal (rec), and passing (pas) mode in the two target size conditions, small and large. Here and in the other figures, the error bars represent standard error (SE). Peak velocity was lower in older than young adults, for small than large targets, and it varied across the three movement modes.

One of the robust features of movement slowness caused by aging is decreased peak velocity. We analyzed peak velocity to assess whether older adults were slower than young adults in the present experiment. The ANOVA results for peak velocity and other studied characteristics are shown in Table 1. All main effects and interactions were significant, except for the three-factor interaction. The mean and standard error (SE) data are shown in Fig. 2. The significantly lower peak velocity in movements of older than young adults confirmed that older adults were slower than young adults in all conditions. The main effect of target size was consistent with the speed-accuracy trade-off, showing that movement speed decreased with decreases in target size. The main effect of movement mode was further investigated with post hoc testing. It was found that peak velocity was the highest during passing movements and the lowest during reciprocal movements, with discrete movements being in between the two other modes. In addition, the significant interactions highlighted that young adults increased peak velocity with increases in target size to a larger extent than older adults. The differences among the three modes were also more pronounced in young than older adults. Finally, the increases in peak velocity during the passing mode were greater for large than small targets.

**Table 1:** Statistical results (F-values and the level of significance).

	Group	Size	Mode	Group × Size	Group × Mode	Size × Mode	Group × Size × Mode
Degrees of Freedom	1, 30	1, 30	2, 60	1, 30	2, 60	2, 60	2, 60
Vpeak	75.9***	234.7***	74.5***	7.5**	29.3***	30.0***	1.1
Primary SM Distance	5.1*	7.3*	137.6***	6.6*	0.6	8.7**	5.6*
SM Incidence, Total	8.5**	83.5***	90.0***	16.3***	0.1	51.2***	0.0
SM Incidence, Type 1	13.6**	27.8***	4.2*	0.0	14.0***	1.4	3.0
SM Incidence, Type 2	5.7*	43.8***	19.1***	2.7	2.0	3.8	2.8
SM Incidence, Type 3	51.2***	102.6***	74.4***	12.7**	31.3***	38.3***	11.5**

* p < 0.05, ** p < 0.01, *** p < 0.001, Vpeak – peak velocity, SM – submovement

### Primary submovement distance

**Figure 3:**
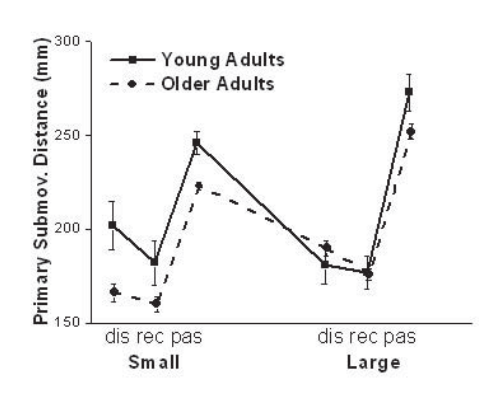
**Distance of primary submovement**. Distance covered in the primary submovement during the discrete (dis), reciprocal (rec), and passing (pas) mode in the two target size conditions, small and large. Primary submovement distance was significantly shorter in older than young adults, specifically during movements to small targets.

Distance covered in the primary submovement was assessed because this characteristic has often been used to support the traditional interpretation of submovements. All main effects and interactions were significant for the primary submovement distance. Fig. 3 clarifies the statistical results. All three main interactions as well as the group by size and size by mode interactions were significant. The major finding that can be inferred from these results is that older adults produced a shorter primary submovement than young adults but this group difference was specifically pronounced during movements to small targets. For large targets, the primary submovement distance was not different between the groups, at least in the discrete and reciprocal mode. This result is consistent with previous studies that reported a shortened primary submovement in older adults, specifically during movements to smaller than to larger targets [2,22,24].

### Total submovement incidence

**Figure 4:**
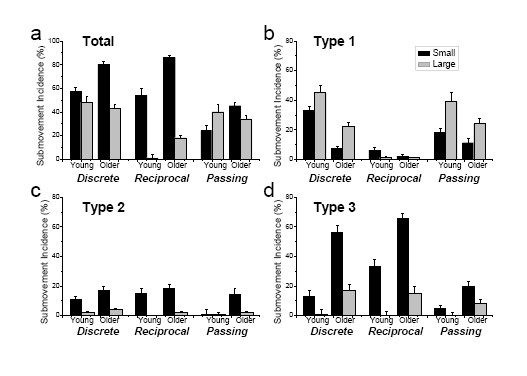
**Submovement incidence**. Total submovement incidences (a) and incidence of type 1, 2 and 3 submovements (b-d) expressed in percentage of the total number of movements in each combination of movement mode (discrete, continuous, and passing) and target size (small and large). The sum of the submovement incidence across the three types in each condition is equal to the total incidence of submovements in this condition. The dependence of submovement incidence on group, movement mode, and target size was specific for each submovement type.

Submovements were found in 40% of all recorded movements in young adults and in 51% of movements in older adults. Fig. 4a shows mean and SE of total submovement incidence (without distinguishing the three submovement types) in each condition and each group. Total submovement incidence depended on each of the three tested factors as revealed by significant main effects of group, target size, and movement mode. On average, the total submovement incidence was greater in older than young adults. However, Fig. 4a shows that this relationship took place predominantly during movements to small and not to large targets. This conclusion was supported by the significant group by size interaction. The group difference during movements to large targets was less straightforward. Although the group by mode and the three-factor interaction were not significant, post hoc testing revealed that in the large-target condition, the differences in submovement incidence between older and young adults was significant during the reciprocal mode (p < 0.001) and not significant during the other two modes. The significant size effect indicates that submovements were more frequent in both groups when the target was small than when it was large. However, Fig. 4a shows that the differences in submovement incidence between the two target sizes were more pronounced during the reciprocal mode than during the other two modes. This observation is consistent with the significant size by mode interaction. The significant mode effect represented the fact revealed in post hoc testing that submovements were more frequent during the discrete mode than during the other two modes.

While the group influence on the total submovement incidence during movements to small targets was consistent with previous findings of the aging effect on submovement production, the effect of aging during movements to large targets depended on movement mode. The complex influence of aging on submovement incidence was clarified by the analysis of submovement incidence conducted separately for each submovement type.

### Submovement incidence by type

The data for each type are shown in Fig. 4b–d, respectively. All three main effects were significant for each of the three types. However, the influence of each factor was different for the different types. Only type 2 and 3 submovements were more frequent in older than in young adults, while the group effect was opposite for type 1 submovement incidence. Type 1 submovements were also remarkable in terms of the effect of target size. These submovements were more frequent during movements to large than to small targets, whereas incidence of submovements of the other two types was in the inverse proportion to the target size. The effect of movement mode was also different across the submovement types. Type 1 submovements were predominantly observed in the discrete and passing but not reciprocal mode. Type 2 submovements were infrequent in all three modes, but specifically in the passing mode. Type 3 submovement incidence was the greatest in discrete movements and the lowest in passing movements with reciprocal movements being in between. These observations are apparent from Fig. 4, and they have also been confirmed in post hoc testing.

### Submovements of type 1

The distinct effect of target size and movement mode on type 1 submovements points to motion termination as the primary source of these submovements. Indeed, these submovements were frequent during the discrete and passing modes that included motion termination and they were rare during the reciprocal mode that did not include motion termination. Also, type 1 submovement incidence increased with increases in target size. This property of type 1 submovements is consistent with the interpretation of them as emergent from motion termination because movements to large targets were faster, and therefore, motion termination and stabilization of the limb at the target would be more likely accompanied with small oscillations than movements to small targets. The factor of movement speed also accounts for the finding that type 1 submovements were less frequent in movements of older adults (that were slower) than in movements of young adults (that were faster). The only significant interaction for type 1 submovement incidence was between group and mode, pointing to a trivial fact that the difference between the two groups observed in the discrete and passing mode disappeared in the reciprocal mode, during which type 1 submovements were almost absent in both groups.

### Submovements of type 2 and 3

In contrast to type 1, incidence of type 2 and 3 submovements increased with decreases in target size. This characteristic is consistent with the interpretation of these submovements as corrective adjustments compensating for target undershooting in the primary submovement. However, at least a portion of these submovements could also be non-corrective. This follows from their appearance in the passing mode after crossing the target, i.e. in the movement stage in which corrective adjustments propelling the limb for further distance were not needed, since the target had already been passed. There are two noteworthy observations with respect to type 2 and 3 submovements in the passing mode. First, incidence of these submovements increased with decreases in target size, as in the other two modes. This observation is important because it shows that the inverse relationship between submovement incidence and target size does not necessarily mean that the submovements are corrective. Second, type 2 and 3 submovements in the passing mode were more frequent in older than young adults. This finding suggests that type 2 and 3 submovements in movements of older adults in the other modes could also have been non-corrective.

Incidence of type 2 and 3 submovements was lower during the passing mode as compared to each of the other two modes. One possible explanation for this difference is that the discrete and reciprocal mode included both corrective and non-corrective submovements. However, the decreased submovement incidence in the passive mode can be accounted for even if we assume that there were no corrective submovements in any modes. For instance, it is discussed in the next section that type 2 and 3 submovements may have represented fluctuations in the velocity profile that emerged with decreases in movement speed. Since passing movements were faster than discrete and reciprocal movements, these fluctuations would emerge less frequently in the passing mode. Also, in the passing mode, the target crossing usually occurred later than peak velocity was achieved. For small targets, the percentage of the deceleration duration elapsed before the target crossing was 33% (± 2.9%) and 32% (± 3.3%) for young and older adults, respectively. This percentage was 13% (± 2.2%) and 3% (± 2.0%) for the two groups during movements to large targets. Thus, only a portion of the deceleration phase was analyzed for the emergence of the secondary submovement in the passing mode, whereas this analysis was performed within the entire deceleration phase in the discrete and reciprocal movements.

To summarize, the results for type 2 and 3 submovements show that at least a part of these submovements could be non-corrective. Although the present results do not exclude a possibility that some of these submovements were corrective, it is also possible that all detected type 2 and 3 submovements were non-corrective. In combination with the results for type 1 submovements, the data show that there is a possibility that the majority of submovements registered in the present experiment were non-corrective.

## Discussion

Previous research has recognized that movements of older adults often include velocity irregularities emerging primarily during the deceleration phase. These irregularities have been interpreted as corrective submovements [2,19-24,36]. The underlying reasoning was that aging-related declines, such as increases in neuromuscular noise, decreases in muscle force, and declines in efficiency of sensory feedback affect accuracy of motion generated by the initial pulse of muscle force. To improve accuracy at the target, older adults decrease the initial force pulse, which results in shortening of the primary submovement and the need for a secondary submovement. The results of the present study question the traditional interpretation of the secondary submovements in older adults. The possibility that secondary submovements may be non-corrective became evident when the three submovement types were analyzed separately from each other. This analysis demonstrated that submovements of each type had specific dependence on movement mode, target size, and subject group.

### Sources of type 1 submovements

The results for type 1 submovements suggest that they emerged predominantly as velocity fluctuations caused by motion termination during which the energy of motion is dissipated and the limb is stabilized at the target. Indeed, these submovements were observed primarily in the discrete and passing mode that included motion termination, and they were rare in the reciprocal mode that did not include motion termination. Furthermore, type 1 submovements were more frequent during movements to large than small targets, which is consistent with the view that these submovements emerged due to motion termination and is not consistent with their interpretation as corrective submovements. The results suggest that movements of older adults are prone to type 1 submovements to a lesser extent than movements of young adults, probably because of the lower movement speed in older adults.

### Sources of type 2 and 3 submovements

Incidence of type 2 submovements was similar in all three modes. These submovements emerged primarily during movements to small targets and they were almost absent during movements to large targets. These characteristics of type 2 submovements were different from characteristics of these submovements documented in our previous studies [25,26]. In those studies, the behaviour of type 2 submovements was similar to that of type 1 submovements, suggesting their emergence from motion termination. For instance, in [25], type 2 submovements were observed predominantly in the discrete and not reciprocal mode, and their incidence was similar for small and larger targets. Similar results for type 2 submovements were obtained in [26] where cyclical movements were examined instead of reciprocal movements. Various reasons can account for the difference between the present and previous studies with respect to type 2 submovements. One possible reason is the ultimate requirement to achieve the target that was used in the present study but not in [25,26]. Also, subjects controlled arm movements while watching motion of the cursor on the computer screen in the present study, whereas movements were performed in natural vision conditions in our previous experiments. Whatever the reasons are, the comparison between the present and previous studies suggests that the sources and behaviour of type 2 submovements can change depending on movement conditions.

What was the source of type 2 submovements in the present study? The inverse dependence on target size and also greater incidence in older than young adults is in agreement with the interpretation of type 2 submovements as corrective accuracy adjustments. However, type 2 submovements were also observed in the portion of passing movements after the target crossing where corrective submovements were unlikely. This finding questions the traditional interpretation of type 2 submovements as corrective adjustments. Furthermore, type 2 submovements in the passing mode were also characterized by the inverse dependence on target size and by the increased frequency in older than young adults, suggesting that these features do not necessarily mean that type 2 submovements are corrective.

The considerations suggesting that type 2 submovements may be non-corrective apply also to type 3 submovements that also emerged more frequently during movements to small than large targets and in older than young adults during all three modes, including the passing mode. The only difference between type 2 and 3 submovements was that type 3 submovements were more frequent in the discrete and reciprocal mode than in the passing mode, and this difference was specifically apparent in older adults. The presence of type 2 and 3 submovements in the passing mode suggests that movements of older adults included non-corrective type 2 and 3 submovements that were more frequent in the small target condition.

A possible consideration against this conclusion could be that subjects performed passing movements as movements to an imaginary target with the given target serving as a via-point. In this case, one can argue that submovements observed after passing the given target were corrective submovements performed with the purpose to achieve the imaginary target. Performance of such submovements is however unlikely. First, passing movements were performed as a sweeping action that included deceleration of motion after the given target had been passed, but it did not include any constraints on the specific location for the movement end. Furthermore, even if there was an imaginary target, performance of corrective submovements with a purpose to accurately achieve this target seems to be implausible. This scenario implies that when the primary submovement undershoots the imaginary target, subjects would make an effort to process feedback to detect this event and to reaccelerate the arm towards the imaginary target, even though there are no limitations on the location and size of this target. Moreover, subjects may not have clear memory of these characteristics hypothetically defined during movement planning. The persistence in achieving the imaginary target could be explained only if the controller dictates that once choosing an imaginary target at the stage of movement planning, motion would be "programmed" not to stop until that target is accurately achieved. Multiple experiments with pointing movements in young adults, including our previous studies [25,26], and in older adults (e.g. [1,24]) suggest that this is not the case. Even during pointing to a physical, clearly visible and well-defined target, motion is often terminated near and not exactly on the target. These considerations show that the production of corrective submovements after the target has been passed is highly implausible.

What could be a source of non-corrective submovements of type 2 and 3? An answer to this question can be suggested if we notice that advanced age and decreases in target size both resulted in decreases in movement speed. Thus, it is possible that type 2 and 3 submovements represented velocity fluctuations that emerged more frequently when movement speed decreased. The hypothesis that fine submovements may be a feature of low movement speed was formulated in [26] where submovements were examined during cyclical movements. It was found that type 3 submovement incidence was significantly influenced by cyclic frequency but not by target size. All findings of the present study with respect to type 2 and 3 submovements are consistent with the interpretation that the emergence of these submovements is associated with decreases in movement speed.

### Possible aging-related declines contributing to type 2 and 3 submovements

The higher submovement incidence in older compared with young adults was attributed primarily to type 3 submovements. Comparison between trends in peak velocity (Fig. 2) and in incidence of type 3 submovements (Fig. 4d) shows that the decreases in target size caused proportional decreases in peak velocity in the two subject groups. However, the effect of target size on type 3 submovement incidence was much stronger in older than in young adults. Thus, decreases in movement speed alone do not account for the dramatic increases in incidence of these submovements in older adults. Different declines in control of muscle activity caused by aging may contribute to frequent type 3 submovements in older adults during slow movements.

First, slow movements are characterized by low acceleration, and therefore, require steady production of low muscle force. The ability to generate smooth muscle force, specifically at low force levels, is decreased by motor unit reorganization observed in aging [37]. This process is represented by reduction in the number of motor units as a result of death of motor neurons in the spinal cord and of increases in the number of muscle fibers innervated by surviving motor neurons [38,39]. The aging-related decline in the ability to maintain smooth generation of muscle force at low levels would result in increased incidence of type 3, and sometimes type 2 submovements in older adults.

Second, co-activation of antagonistic muscles may contribute to the emergence of type 2 and 3 submovements by causing random fluctuations in the resultant muscle force. Findings consistent with this assumption are that muscle co-activation increases with decreases in target size [40], and it is higher in older than young adults [21,36].

Finally, a hypothesis that pointing accuracy is achieved via regulation of arm stiffness [40-42] also predicts submovements during slow movements. According to this hypothesis, increased stiffness resists movement perturbations that may emerge due to noise in the neuromuscular control signals. Greater limb stiffness slows motion down and increases the signal-to-noise ratio of forces that drive the limb to the target, which may cause velocity fluctuations in the form of type 2 and 3 submovements. Variability of motor output caused by increased stiffness may be enhanced in movements of older adults who have difficulties with regulation of muscle force [43].

## Conclusion

While previous studies have interpreted submovements as corrective adjustments that contribute to slowness in aging, the findings of the present study demonstrate a possibility that many submovements are not corrective in both young and older adults. Two viable sources of non-corrective submovements are suggested by the results of the experiment. Type 1 submovements emerged during motion termination and the stabilization of the arm performed in the discrete and passing mode. Although the nature of type 2 and 3 non-corrective submovements requires further investigation, it is plausible that they represented velocity fluctuations that became more pronounced with decreases in movement speed. While our data do not exclude that some submovements were corrective, they show a distinct possibility that the majority of submovements were non-corrective. If this is the case, the long-held interpretation that submovements are one of the major reasons for movement slowness in older adults would need to be reconsidered. Frequent submovements in older adults would rather be a consequence of movement slowness observed in aging.

## Competing interests

The authors declare that they have no competing interests.

## Authors' contributions

LF carried out data analysis and has been critically involved in the manuscript preparation. GL has made important contribution in the design of the experiment, collected data, and has been involved in revising the manuscript. ND has made critical contribution to development of conception and design of the study, data analysis, and manuscript preparation. All authors read and approved the final manuscript.
